# Clinical-like cryotherapy improves footprint patterns and reduces synovial inflammation in a rat model of post-traumatic knee osteoarthritis

**DOI:** 10.1038/s41598-019-50958-8

**Published:** 2019-10-10

**Authors:** Germanna Medeiros Barbosa, Jonathan Emanuel Cunha, Thiago Mattar Cunha, Lizandra Botaro Martinho, Paula Aiello Tomé Souza Castro, Francisco Fábio Bezerra Oliveira, Fernando Queiróz Cunha, Fernando Silva Ramalho, Tania Fátima Salvini

**Affiliations:** 1 Department of Physical Therapy, Federal University of São Carlos, São Carlos, SP Brazil; 2 Department of Pharmacology, University of São Paulo, Ribeirão Preto, SP Brazil; 3 Department of Pathology and Forensic Medicine, University of São Paulo, Ribeirão Preto, SP Brazil

**Keywords:** Interleukins, Osteoarthritis, Cartilage

## Abstract

Cryotherapy is a non-pharmacological treatment commonly used to control inflammation and improve function after acute traumas. However, there are no definitive findings about its effects on chronic joint diseases such as knee osteoarthritis (KOA). The aim of this study was to investigate the effects of clinical-like cryotherapy on functional impairment and synovial inflammation in a rat model of KOA generated by anterior cruciate ligament transection (ACLT). Thirty-two male *Wistar* rats were randomly divided into four groups (n = 8/group): Control, KOA, KOA + Cryotherapy and KOA + Placebo. The last two groups were submitted to the relevant interventions twice a day for five days (61 to 65), with each session lasting 20 min. Gait test, skin temperature, thermal response threshold and joint swelling were assessed in all groups before ACLT surgery, and pre (60^th^ day) and post (66^th^ day) intervention protocols. On day 66, the animals were euthanized and exsanguinated to remove the synovial membrane for histopathological examination and synovial fluid to determine the leukocyte count and cytokine concentration. After the intervention period (66^th^ day), footprint area only increased in the KOA + Cryotherapy group (*P* = 0.004; 14%) when compared to KOA and KOA + Placebo, but did not differ from controls. Cryotherapy lowered the synovial fluid leukocyte count (*P* < 0.0001; ≥95.0%) and cytokine concentration (*P* < 0.0001; ≥55%) when compared to the KOA and Placebo groups. Synovial score and synovial fibrosis did not differ in the KOA groups. In conclusion, footprint patterns improved in rats with ACLT-induced KOA as a result of clinical-like cryotherapy, which also lowered the synovial fluid leukocyte count and inflammatory cytokine concentration in these rats.

## Introduction

Knee osteoarthritis (KOA), the most frequent degenerative articular disorder in adults and seniors, is considered one of the primary causes of pain and functional disability^1,2^. A series of risk factors are associated with KOA^3^. It is known that a ruptured or reconstructed anterior cruciate ligament increases the likelihood of KOA progression^4^. Animal models of tibiofemoral instability, such as anterior cruciate ligament transection (ACLT), have been used to induce changes similar to those of post-traumatic and chronic KOA in humans^5,6^. A recent study showed that ACLT-induced KOA in rats promotes neuromuscular junction remodeling and atrophy in the quadriceps and tibialis anterior muscles, associated with inflammatory signs, gait changes, and alterations in muscle gene and protein expression^7^. Post-trauma biochemical and biomechanical changes alter the metabolic pattern of chondrocytes, which activate adaptive responses, including pro-inflammatory cytokines^8,9^. Among these cytokines, interleukin (IL)-1β, IL-6 and the tumor necrosis factor (TNF)-α stand out in the pathophysiology of KOA, due to their catabolic and destructive effects on joints^8^. These changes are progressive and frequently identified by radiographic and symptomatological signs^10^.

The control of pro-inflammatory cytokines has been considered a therapeutic pharmacological approach in the treatment of KOA^11^. However, like other pharmacological therapies, anticytokine drugs exhibit potential iatrogenic effects^12,13^, favoring the search for complementary treatments. Cryotherapy is a non-pharmacological resource used to control inflammation and improves the clinical condition after acute musculoskeletal trauma^14–16^. Moreover, it is low-cost, relatively safe and easy to use^17^. Although cryotherapy is widely used to control pain and inflammation following acute trauma, with strong evidence of its beneficial effects^18,19^, little is known about its impact on chronic joint injury^20^. A recent review showed that although some clinical guidelines recommend cryotherapy to manage KOA symptoms, there is insufficient evidence regarding its use for this population^21^. Few animal studies have investigated the issue, with heterogenic protocols (physical agents used, application time and periodicity) focused primarily on the immediate responses of cryotherapy in animal models of knee arthritis^22–26^. More recently, it was demonstrated that local cryotherapy applied for 14 consecutive days has a local and systematic anti-inflammatory effect on adjuvant-induced arthritis, mainly through IL-6/IL-17 pathway inhibition, independent of TNF-α^27^. Although this is a relevant finding, the study was conducted in an animal model of rheumatoid arthritis, with potentially destructive immunological effects on joints^28^, and the cryotherapy protocol did not follow the clinical recommendations for musculoskeletal disorders^21,29^. We found no previous studies that assessed the effect of cryotherapy in an animal model of KOA, with similar characteristics to those observed in humans. A possible beneficial effect of cryotherapy in controlling KOA inflammation in an animal model could provide new scientific evidence for its clinical use. The aim of this study was to investigate the effects of clinical-like cryotherapy on functional impairment and synovial inflammation in a rat model of KOA generated by ACLT. Our hypothesis was that cryotherapy would improve the gait function and reduce inflammatory signs of the animals.

## Results

### Gait analysis

**Figure 1:**
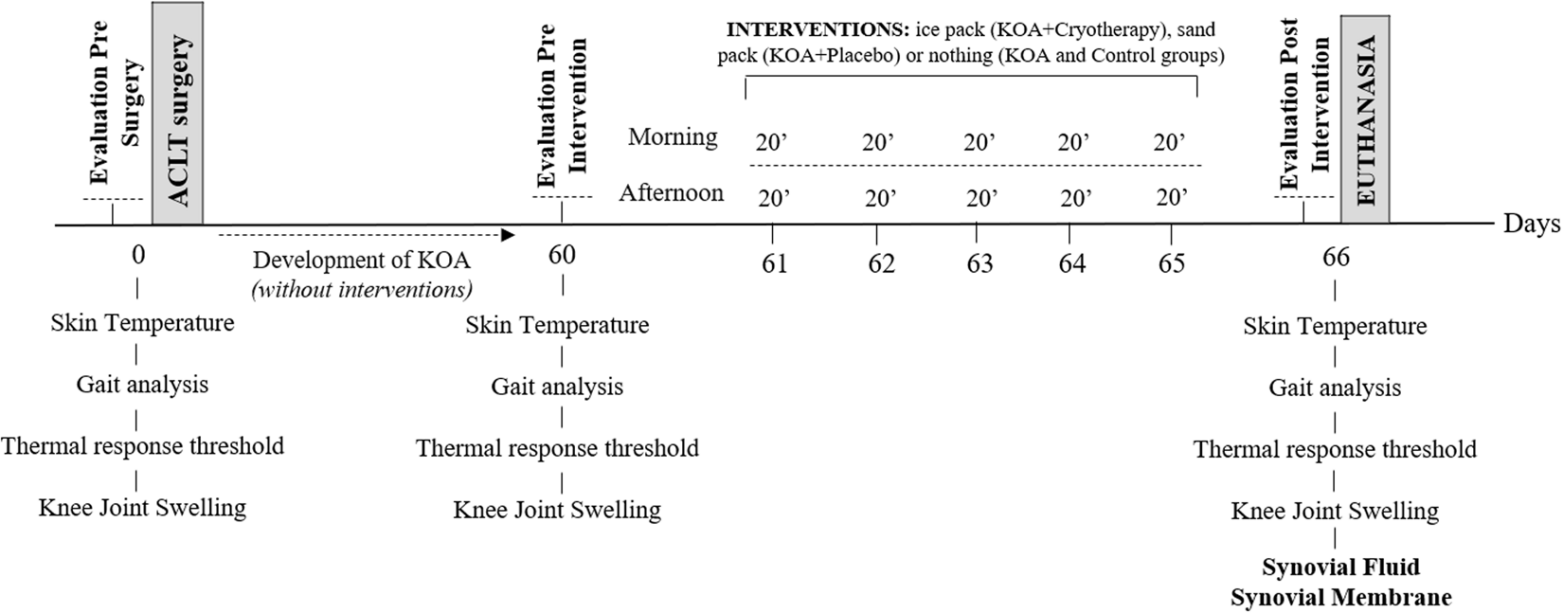
Experimental procedures. The animals in both groups received two 20-min interventions per day, one in the morning and one in the afternoon. KOA – knee osteoarthritis, ACLT – anterior cruciate ligament transection.

**Figure 2:**
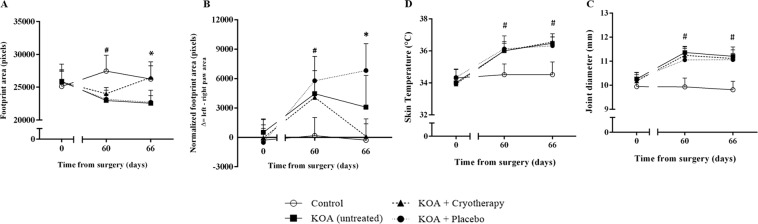
Footprint area (**A**), normalized footprint area (**B**) skin temperature (**C**), joint diameter (**D**) at baseline (0 day, pre-surgery), pre- (60^th^ day) and post-intervention (66^th^ day) protocol in all groups. KOA: knee surgery (anterior cruciate ligament transection). Data are expressed as mean ± SD (n = 8/group). ^#^*P* < 0.01: Control group *vs* KOA, KOA + Cryotherapy and KOA + Placebo groups; *P < 0.05: both Control and KOA + Cryotherapy groups *vs* KOA and KOA + Placebo groups.

Sixty days after ACLT (Fig. 1), the KOA, KOA + Cryotherapy and KOA + Placebo groups showed a lower footprint area compared to Control group [mean difference: −4478 pixels (−16.3%), 95% CI: −6831, −2124, *P* < 0.0001; mean difference: −3445 pixels (−12.5%), 95% CI: −5798, −1092, *P* = 0.002; mean difference: −4280 pixels (−15.6%), 95% CI: −6633, −1926, *P* < 0.0001, respectively]. With respect to the KOA groups, footprint area only increased in the group submitted to cryotherapy [KOA, mean difference: 3936 pixels (15%), 95% CI: 1021, 6850, *P* = 0.004; KOA + Placebo, mean difference: 3779 pixels (14%), 95% CI: 864, 6694, *P* = 0.006] after interventions, with no alterations in relation to controls (95% CI: −2671, 3158; *P* > 0.05) [Fig. 2(A)]. In order to estimate that weight-bearing balance between the two limbs was independent of animal size and walking velocity, we normalized the data related to the contralateral limb (pixel size of the left minus the right footprint area). On the 60^th^ day after ACLT, the KOA groups showed more weight-bearing in the left (unaffected) paw when compared to controls [KOA, mean difference: 4284 pixels (96.2%), 95% CI: 3114, 5796; *P* < 0.001; KOA + cryotherapy, mean difference: 3915 pixels (95.8%), 95% CI: 1367, 6803; *P* = 0.02; KOA + Placebo, mean difference: 5606 pixels (97.0%), 95% CI: 8264, 3064, *P* < 0.001]. Among KOA groups, only the Cryotherapy group walked with more symmetrical weight-bearing between paws on the 66^th^ day [KOA, mean difference: 2972 (96.7%); 95% CI: 180, 6329; *P* = 0.04; KOA + Placebo, mean difference: 6712 (98.5%), 95% CI: 4071, 9559; *P* < 0.001)], with no differences in relation to controls (95% CI: −1967, 1384; *P* > 0.05), Fig. 2(B). There were no changes in the other variables related to gait analysis (Supplementary Appendix I).

### Knee skin temperature

On the 60^th^ day, skin temperature was higher in the KOA groups when compared to Controls (KOA, mean difference: 1.5°, 95% CI: −2.4, −0.6; KOA + Cryotherapy, mean difference: 1.5 °C, 95% CI: −2.4, −0.6; KOA + Placebo, mean difference: 1.6 °C, 95% CI: −2.5, −0.7, *P* < 0.0001). These differences continued on the 66^th^ day, irrespective of the intervention (KOA, mean difference: 1.9°, 95% CI: −2.8, −1.1; KOA + Cryotherapy, mean difference: 2.0 °C, 95% CI: −2.9, −1.2; KOA + Placebo, mean difference: 1.8 °C, 95% CI: −2.7, −0.9, *P* < 0.0001) [Fig. 2(C)].

### Knee joint swelling

On the 60^th^ day, knee joint diameter was larger in the KOA groups compared to the Control group (KOA, mean difference: 1.4 mm, 95% CI: −2.0, −0.9; KOA + Cryotherapy, mean difference: 1.3 mm, 95% CI: −1.8, −0.8; KOA + Placebo, mean difference: 1.1 mm, 95% CI: −1.6, −0.6, *P* < 0.0001). These differences were unchanged on the 66^th^ day, regardless of the intervention (KOA, mean difference: 1.4 mm, 95% CI: −1.9, −0.9; KOA + Cryotherapy, mean difference: 1.3 mm, 95% CI: −1.8, −0.8; KOA + Placebo, mean difference: 1.1 mm, 95% CI: −1.8, −0.7; *P* < 0.0001) [Fig. 2(D)].

### Thermal response threshold

There were no intergroup differences in the thermal response threshold (*P* = 0.184; Supplementary Appendix II).

### *In Vivo* Leukocyte migration to synovial fluid

**Figure 3:**
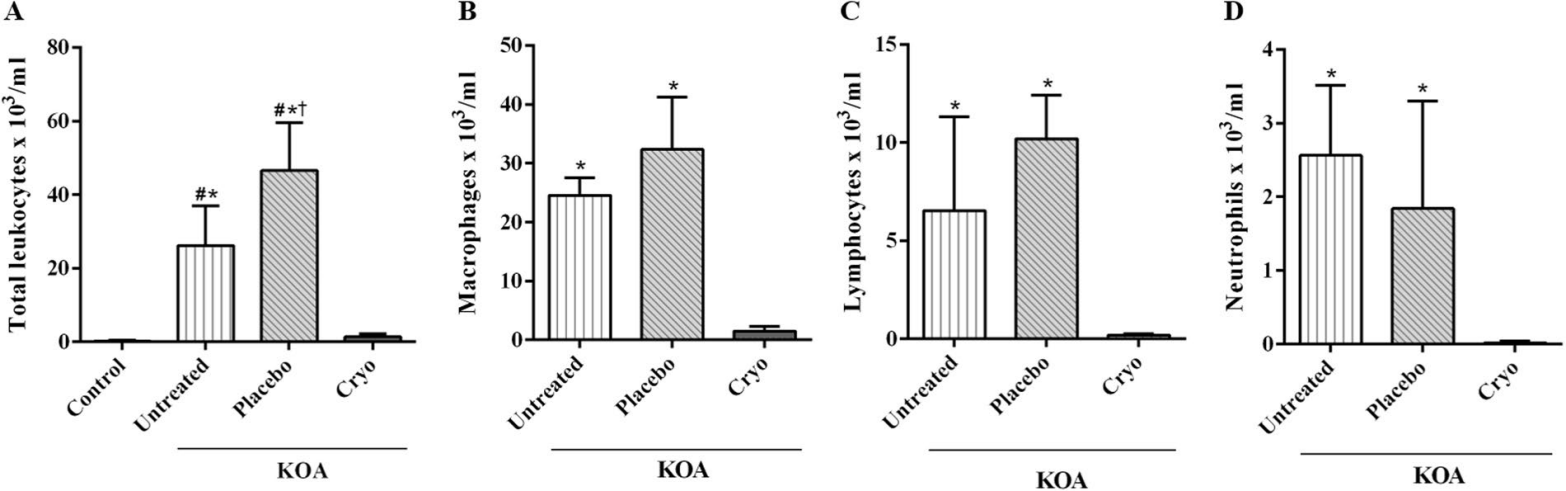
Number of cells in knee synovial fluid. Total number of leukocytes (**A**) and differential macrophage (**B**), lymphocyte (**C**) and neutrophil count. (**D**) KOA: knee surgery (anterior cruciate ligament transection); Cryo: cryotherapy. Data are expressed as mean ± SD (n = 7–8/group). ^#^*P* < 0.05 vs Control group; ^†^*P* < 0.05 vs KOA group; *P < 0.05 vs KOA + Cryotherapy group.

There was a significant reduction in the number of leukocytes in the KOA + Cryotherapy group compared to KOA [mean difference: −0.65 × 10^3^/ml (−95.0%), 95% CI: −1.01, −0.29, *P* < 0.0001] and KOA + Placebo [mean difference: −1.05 × 10^3^/ml (−97.0%), 95% CI: −1.41, −0.69, *P* < 0.0001] groups, but with no difference compared to Control group (95% CI: −0.34,0.38, *P* = 0.99). The KOA and KOA + Placebo groups contained more leukocytes than the Control group [mean difference: 0.67 × 10^3^/ml (99.1%), 95% CI: 0.31, 1.03, *P* < 0.0001; mean difference: 1.07 × 10^3^/ml (99.5%), 95% CI: 0.70, 1.43, *P* < 0.0001, respectively]. The number of leukocytes was 43.1% higher in the KOA + Placebo group when compared to the KOA group (mean difference: 0.39 × 10^3^/ml, 95% CI: 0.03, 0.76, *P* = 0.028) [Fig. 3(A)].

The differential leukocytes count [Fig. 3(B–D)] showed a significant decline in the number of cells in the KOA + Cryotherapy group, when compared to the KOA group [macrophages, mean difference: −23.07 × 10^3^/ml (−94.0%), 95% CI: −33.19,−12.95, *P* < 0.0001; neutrophils, mean difference: −2.53 × 10^3^/ml (−99.3%), 95% CI: −4.45, −0.62, *P* = 0.008; lymphocytes, mean difference: −6.34 × 10^3^/ml (−97.1%), 95% CI: −11.67, −1.01, *P* = 0.02] and KOA + Placebo [macrophages, mean difference: −30.87 × 10^3^/ml (−95.0%), 95% CI: −40.41, −21.33, *P* < 0.0001; neutrophils, mean difference: −1.82 × 10^3^/ml (−99.0%), 95% CI: −3.55, −0.09, *P* = 0.039; lymphocytes, mean difference: −10.02 × 10^3^/ml (−98.1%), 95% CI: −15.34, −4.68, *P* = 0.001].

### Knee joint synovial fluid cytokines

**Figure 4:**
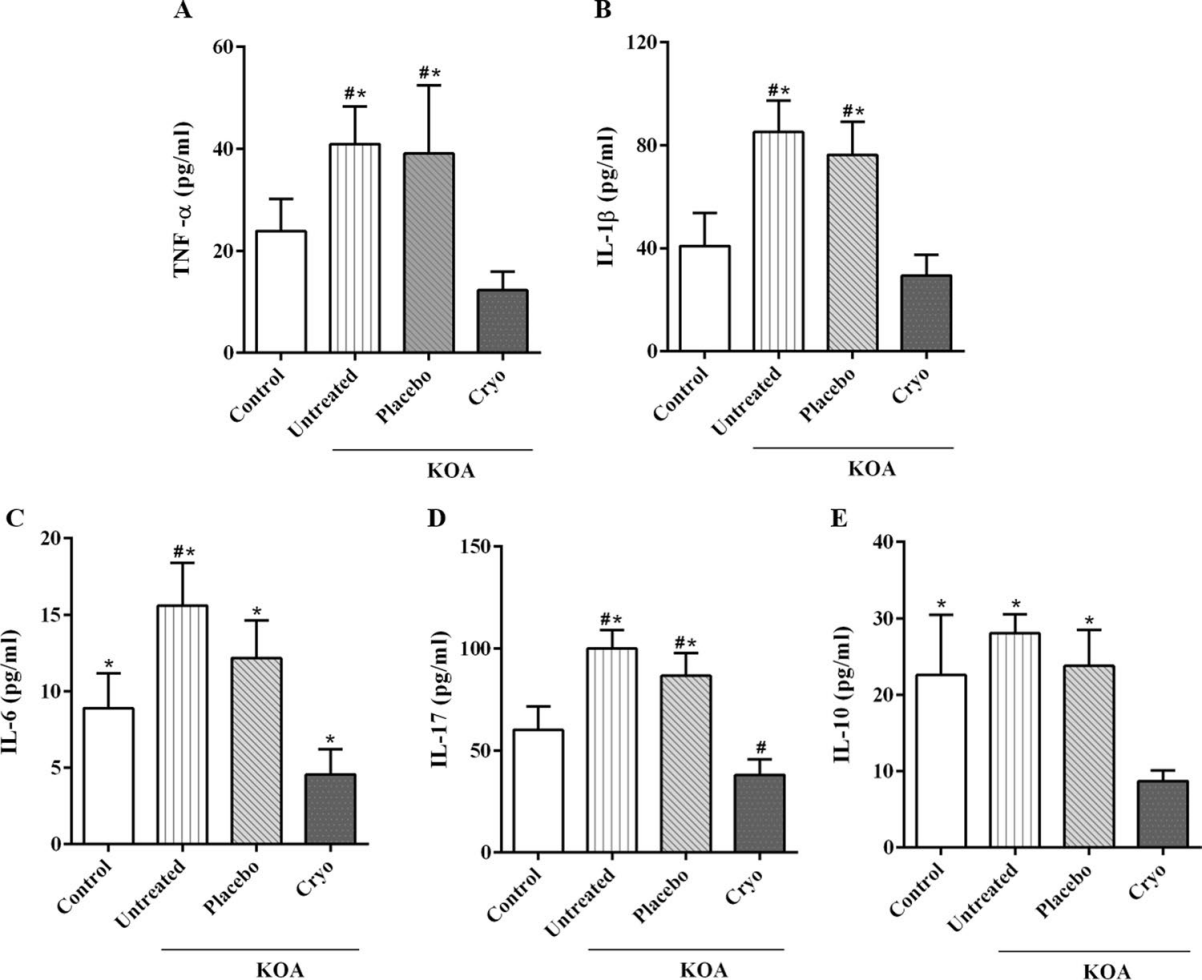
Cytokine concentration in knee synovial fluid. (**A**) TNF-α; (**B**) IL1-β; (**C**) IL-6; (**D**) IL-17; (**E**) IL-10. KOA: knee surgery (anterior cruciate ligament transection); Cryo: cryotherapy. Data are expressed as mean ± SD (n = 7–8/group). ^#^*P* < 0.05 vs Control group; *P < 0.05 vs KOA + Cryotherapy group.

The KOA + Cryotherapy group contained a lower concentration of cytokines compared to the KOA [IL-1β, mean difference: −55.70 pg/ml (−65.3%), 95% CI: −73.84,−37.48; TNF-α, mean difference: −28.65 pg/ml (−70.0%), 95% CI: −41.13,−16.17; IL-6, mean difference: −11.02 pg/ml (−70.7%), 95% CI: −14.46, −7.59; IL-17, mean difference: −62.51 pg/ml (−61.9%), 95% CI: −77.14, −47.88; IL-10, mean difference: −19.34 pg/ml (−68.9%), 95% CI: −26.42, −12.26; P < 0.0001] and KOA + Placebo groups [IL-1β, mean difference: −46.8 pg/ml (−61.3%), 95% CI: −64.9, −28.6; TNF-α, mean difference: −26.8 pg/ml (−68.6%), 95% CI: −39.3, −14.4; IL-6, mean difference: −7.6 pg/ml (−62.5%), 95% CI: −11.0, −4.2; IL-17, mean difference: −48.5 (−55.9%), 95% CI: −63.1, −33.9; IL-10, mean difference: −15.1 (−63.4%), 95% CI: −22.1, −7.9; *P* < 0.0001], Fig. 4. The KOA + Cryotherapy group also showed lower concentrations of IL-6 [mean difference: −11.0 pg/ml (−48.7%), 95% CI: −14.5, −7.6; *P* < 0.0001], IL-17 [mean difference: −48.5 pg/ml (−36.6%), 95% CI: −63.8, −33.3; *P* < 0.0001] and IL-10 [mean difference: −15.1 pg/ml (−61.4%), 95% CI: −22.1, −7.9; *P* < 0.0001] compared to Control group (Fig. 3C–E). The KOA group exhibited a higher concentration of IL-1β [mean difference: 44.21 pg/ml (51.9%), 95% CI: 26.7, 61.7; *P* < 0.0001]; TNF-α [mean difference: 17.1 pg/ml (41.7%), 95% CI: 4.6, 29.5; *P* = 0.005] and IL-17 [mean difference: 40.5 pg/ml (60.1%), 95% CI: 25.8, 55.1; *P* < 0.0001] compared to Control group [Fig. 4(A),(B),(D)]. The KOA + Placebo had a higher concentration of cytokines [IL-1β, mean difference: 35.3 pg/ml (46.3%), 95% CI: 17.9, 52.8, *P* < 0.0001; TNF-α, mean difference: 15.2 pg/ml (38.9%), 95% CI: 2.7, 27.7, *P* = 0.01; IL-17, mean difference: 26.5 pg/ml (30.55%), 95% CI: 11.8, 41.1, *P* < 0.0001] compared to Control group [Fig. 4(A,B,D)].

### Histopathological assessment of synovitis

**Figure 5:**
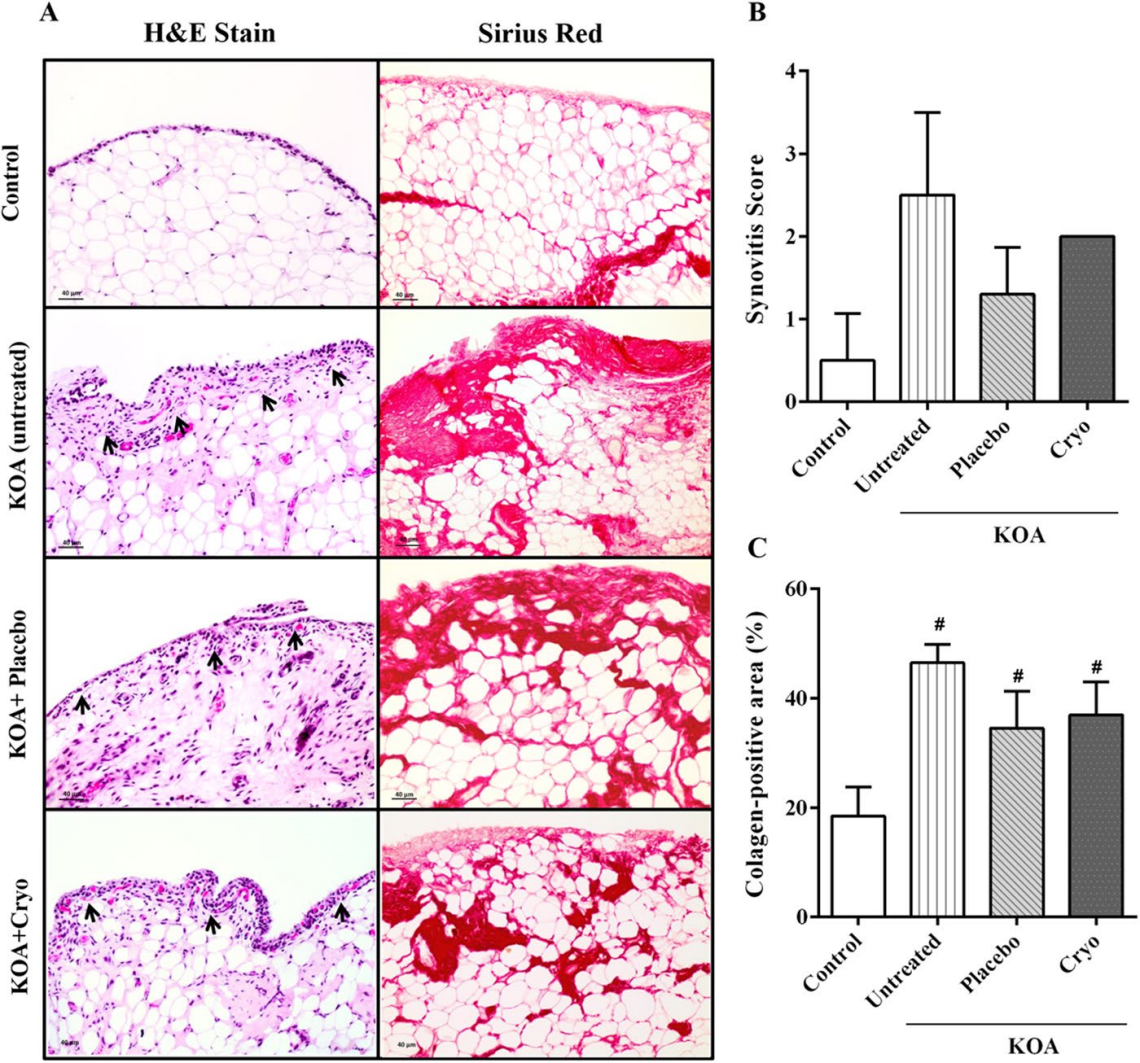
Histopathological assessment of the synovial membrane. Representative photomicrographs of synovial membrane sections stained with Hematoxiline & Eosine (H&E) and Sirius Red in the control, KOA, KOA + Cryotherapy ice and KOA + Placebo groups (Scale bar: 40 µm; 200x magnification) (**A**). Graphic representation of Synovitis Score (**B**) and Fibrosis (**C**) in the Control, KOA + Cryotherapy and KOA + Placebo groups. The arrows indicate the base of the synovial membrane and show its thickness. KOA: knee surgery (anterior cruciate ligament transection); Cryo: cryotherapy. Data are expressed as mean ± SD (n = 3–5/group); ^#^*P* < 0.05 vs Control group.

There was no change in the synovial membrane of the KOA (*P* = 0.017), KOA + Placebo (*P* = 0.115) and KOA + Cryotherapy (*P* = 0.013) groups compared to Control group [Fig. 5(A,B)]. With respect to synovial membrane fibrosis, [Fig. 5(A,C)], KOA groups displayed a large amount of collagen compared to the Control group [KOA, mean difference: −28.0%, 95% CI: −39.5, −16.6; *P* ≤ 0.0001; KOA + Placebo, mean difference: −16.1%, 95% CI: −28,4, −3,7; P = 0.01; KOA + Cryotherapy, mean difference: −18.5%, 95% CI: −29.9, −7.1; *P* = 0.02]. Moreover, there was no difference in synovitis or fibrosis score between KOA groups [(Fig. 5(B,C); *P* > 0.05].

## Discussion

Our results show, for the first time, that clinical-like cryotherapy is a beneficial intervention for ACLT-induced KOA, since it improves footprint patterns, and its effects are mediated by downregulation of joint synovial inflammation. The reduced inflammatory process observed in the Cryotherapy group due to lower leukocyte migration to the joint cavity indicates a beneficial effect on the control of synovial inflammation. These findings demonstrate the potential of cryotherapy as a non-pharmacological treatment for joint inflammation in KOA.

Although the anti-inflammatory effects of cryotherapy are little studied in animal models of arthritis^30^, a number of action mechanisms have been proposed to explain its effects on reducing the inflammatory process in joints. A single long-term ice-pack application (4 hours) decreased leukocyte phagocytosis in the synovial fluid of a dog’s knee with urate crystal-induced synovitis. The effects were attributed to increased viscosity in the synovial fluid, which may have hindered leukocyte movement to the crystals^23^. In another study, ice packs (30 minutes, once a day for 10 days) reduced cell infiltrate and synovial hyperplasia in a rabbit zymosan-induced arthritis model^31^. In a traumatic model, pigs with radiocarpal ligament sprain experienced a decline in the number of leukocytes after two applications of crushed-ice packs (20 min each)^26^. In these studies, synovitis and inflammation were suppressed by lowering intra-articular temperature. According to previous studies, the enzymatic activity responsible for matrix degradation (i.e. collagenases) decreases at lower temperatures^22,32^. Although we did not measure intra-articular temperature, these mechanisms likely also occurred, contributing to the decline in joint inflammation. Another possible mechanism associated with cryotherapy is a decrease in the local metabolism, which was demonstrated in an earlier investigation^33^, causing less cellular infiltrate in the synovial membrane and, consequently, less activation of inflammatory mediators.

It is known that pro-inflammatory cytokines, such as IL1-β and TNF-α, exert catabolic action and contribute in a number of ways to joint degeneration in KOA, including proteinase activation and suppression of type II collagen, one of the main components of the extracellular matrix^8,9^. Other cytokines, such as IL-17, act as a critical mediator in neutrophil recruitment, migration and activation^34^. According to our results, cryotherapy was effective in reducing (≥55%) pro-inflammatory cytokine levels in the synovial fluid. An earlier study on cryotherapy applied to arthritic rat paws (30 minutes, twice a day, for 14 days) showed local and systemic anti-inflammatory effects, which were mediated primarily by genetic and protein expression of IL-6 and IL-17, independent of TNF-α^27^. Studies conducted in different pathological and physiological conditions related the beneficial effects of cryotherapy to NF-kβ- dependent gene inhibition of IL-1β, TNF-α and IL-6^30,35^. These pathways are also involved in the effects of cryotherapy observed in the present study.

Despite the significant decline in the inflammatory process observed in the KOA group submitted to cryotherapy, there was no improvement in the histopathological signs of their synovial membrane. This result shows that the experimental model used induces KOA, and that the signs of the disease in the synovial membrane are unchanged by cryotherapy.

In relation to gait analysis, the larger footprint area affected in the KOA + Cryotherapy compared to KOA and KOA + Placebo groups, and the similar findings to Controls, indicates more homogeneous weight bearing, favoring a normal gait pattern. The results of the present study with the KOA model in rats were similar to those of previous research^36^, which also reported a decline in the footprint area of mice after four and eight weeks of ACLT. Cold-induced analgesia directly affects gait control, decreased nociceptor excitability thresholds and nerve conduction velocities^37^. A decline in nociceptive information transmitted through primary afferents to the spinal cord would result in fewer behavioral signs and reduced neuronal activity of dorsal horn neurons, prompting a reduction of the expanded receptive field, which may impact gait responsiveness^25^. In contrast to that observed in patients with KOA^38,39^, we found no changes in the other variables related to gait analysis in all groups, such as stride length and width. Although our results corroborate with previous findings in rats^36,40,41^, we did not control the gait speed of the animals, an important covariate for nearly all gait parameters. This can be measured in future studies by recording of the time taken (in seconds) to reach the dark chamber (distance traveled divided by the time it took to cover this distance)^42^, statistical models^43^, speed control with treadmill^44,45^ or by using modern high-speed videography (i.e. catwalk), which is more likely to provide a robust analysis of spatial parameters^46^.

Finally, the chronicity characteristics of KOA may explain the absence of a cryotherapy effect on both skin temperature and swelling of the knee joint. There were also no changes in thermal hyperalgesia in the KOA model, which is supported by previous studies of the post-traumatic KOA model^47,48^. Behavioral changes commonly reported in the literature involve the assessment of other parameters, such as gait adaptations, mechanical hyperalgesia, mechanical allodynia and weight-bearing asymmetries.

One of the limitations of this study was using the footprint test to evaluate gait patterns, since it cannot accurately assess the velocity of the animal. Another weakness was the lack of a sham group for the surgical procedure (knee surgery without ACLT); future research should include a sham group for comparison purposes.

## Materials and Methods

The experimental protocol was in accordance with the National Guide for the Care and Use of Laboratory Animals (National Research Council, 1996)^49^. The Federal University of São Carlos Ethics Committee approved the experimental procedures (number 7949291116/2017) and the study was conducted by trained professionals blinded to the identity of the experimental groups^49^. A priori sample calculation was performed using G*Power (version 3.1; University of Trier, Trier, Germany)^50^. Based on a previously performed pilot study (n = 10), it was adopted an α = 0.05, power (1-β) = 0.95, correlation coefficient = 0.5 and effect size = 0.37. Gait test and Leukocyte migration were used in the calculation. Therefore, it was estimated that a total of 32 animals (8 per group), would be an adequate sample size.

### Experimental design

The animals analyzed were two-month-old male *Wistar* rats (*Rattus norvegicus*, n = 32; 297 ± 25 g), housed at 24 °C ± 1 °C (three per cage) under pathogen-free conditions in a reverse light cycle (12/12 light/dark) and given ad libitum access to standard rat chow and water. A computer program (www.random.org) was used to randomly divide the animals into four groups (n = 8 per group): Control (composed of naïve animals not submitted to surgery or intervention); ACLT knee surgery (KOA, untreated group); ACLT knee surgery and an ice pack (KOA + Cryotherapy), and ACLT knee surgery with a sand pack similar to the ice pack (KOA + Placebo)^49^. The groups were assessed one day prior to ACLT surgery and 60 days after the procedure, performing the least stressful tests firsts and progressing to the most stressful ones, as follows: skin temperature, gait test, thermal response threshold and swelling. The interventions (ice pack or sand pack) were then carried out in the the KOA + Cryotherapy and KOA + Placebo groups twice a day for five days (61 to 65), With each session lasting 20 min. All four groups were assessed day 66, and the animals were euthanized and exsanguinated to remove the synovial membrane and synovial fluid (Fig. 1).

### KOA induction

We used an adapted ACLT-induced KOA model that causes joint changes in rats similar to those observed in humans^5^. Briefly, the animals were anesthetized with an intraperitoneal injection (12 mg/kg Xylazine and 95 mg/kg Ketamine) and the right knee was shaved and prepared using an iodine solution. Next, a medial longitudinal parapatellar incision was made and joint capsule injury and ACLT were induced with ophthalmic scissors. The anterior drawer test (free anterior displacement of the tibia in relation to the femur) was performed to confirm ACLT. After the skin was sutured, the animals were returned to their cages and paracetamol (13.5 mg/100 mL) was added to their drinking water for the first 48 hours as postoperative analgesia^5^. In this model we previously described a higher Mankin histological score in the KOA group (60 days after ACLT) when compared to controls (naïve animals)^7^.

### Gait test

Gait analysis was conducted using the paw print test as previously performed in an ACLT animal model^36^. The hind paws of the rats were brushed with ink. Next, the animals were allowed to run on a 60 cm-long, 7 cm-wide track covered with white paper. A dark chamber was placed at the end of the track to entice the rats. Upon completion of the test, the paper was scanned at 300 dpi. The measurement around the right paw was defined as footprint area (pixels), the distance between the first and fifth toe as paw width (cm), the distance between two steps of the same hind paw as stride length (cm), the horizontal distance between the left and right paw as the base (cm), the distance between the third toe and the heel as paw length (cm), and the paw angle as the angle between the fifth toe and the calcaneus and a horizontal line (°).

### Skin temperature

In accordance with the criteria for acquiring thermal images^51^, the animals were acclimated in a dark room (15 min; 23 °C ± 1). Thermography was used to measure the skin temperature of the right knee in all groups, using a FLIR T420 infrared thermal camera (FLIR Systems®, USA), attached to a tripod placed 50 cm from the animal’s knee. The images were analyzed in FLIR Tools software, and the results expressed in °C. A pilot study (n = 8 rats) was conducted to determine skin cooling in the KOA + Cryotherapy group immediately after a single session. The knee skin temperature decreased 26.8 ± 0.4 °C, from 36.9 ± 0.7 °C to 10.1 ± 1.5 °C.

### Knee joint swelling

Knee joint thickness was measured with the animals in the supine position under anesthesia (2 ml/mlO_2_; 1.5% isoflurane)^52^, using a digital caliper (Fisher Scientific, 150 mm, USA), positioned on the medial and lateral femoral condyles, at the knee joint interline level^7^. The mean of two measures was used and the data expressed in millimeters (mm).

### Thermal response threshold

The animals were placed on a hotplate (Insight® Equipamentos Ltda, Brasil) at 52 °C (51.8–52.4 °C)^53^. The latency period for paw response (jumping, shaking, or licking) was considered the response time (seconds), in a single repetition. The maximum time an animal remained on the hotplate was 25 seconds.

### Protocol reliability

The intraclass correlation coefficient (ICC_1,2_) and standard error of measurement (SEM) were tested for thermography analysis (A) and joint swelling (B) in eight rats before surgery. The rats were re-evaluated 48 hours after the first assessment. Both variables exhibited excellent reliability: A) ICC = 0.92, SEM = 0.20 °C; B) ICC = 0.94; SEM = 0.28 mm.

### Cryotherapy and Placebo interventions

Interventions in the KOA + Cryotherapy (20 g pack of crushed ice) and KOA + Placebo groups (20 g sand pack) were carried out twice a day in the laboratory, approximately 4 hours apart. Each intervention lasted 20 minutes^21^ and was applied under anesthesia (2 ml/mlO_2_; 1.5% isoflurane)^52^, with the animals in the supine position with raised paw (hip joint ± 45°). The ice and sand packs were placed around the knee, using an elastic band for compression. The cryotherapy protocol followed the clinical recommendations for the management of musculoskeletal injuries, according to PRICE protocol (Protection, Rest, Ice, Compression and Elevation)^29^. Despite the absence of intervention in the Control and KOA groups, they were submitted to the same anesthesia applied to the intervention groups.

### Synovial fluid collection

The animals were anesthetized (240 mg/kg ketamine and 60 mg/kg xylazine; i.p.) and exsanguinated^54,55^. This last procedure was carried out to minimize the possibility of blood contamination in synovial fluid. The skin and right knee joint ligaments were removed, and the synovial cavity was washed twice with 200 µL of phosphate-buffered saline (PBS) containing 10 mM ethylenediaminetetraacetic acid (EDTA). This joint lavage fluid was used for cell counting, leukocyte differential counting and cytokine level determination^55^.

### *In Vivo* leukocyte migration

Leukocyte migration was determined using synovial fluid, as previously described^56^. The joint cavities were washed twice with 5 μL of PBS containing 1 mM EDTA and then diluted to a final volume of 50 μL with PBS/EDTA to evaluate leukocyte migration at the established time. The leukocytes were counted in a Neubauer chamber diluted in Turk’s solution. The results were expressed as the number of leucocytes per joint cavity.

### Differential leukocyte count

For differential count, aliquots of joint lavage fluid were removed and centrifuged at 1,500 rpm for 10 min at 4 °C. The supernatant was stored at −80 °C for subsequent analysis (cytokine determination), and the cell pellet was resuspended in 200 µL of as PBS plus EDTA solution. Differential count slides were prepared using an aliquot of the washed joint fluid (50 μl) subjected to cytocentrifugation at 1,500 rpm for 10 min. The slides were then mounted, fixed for 4 min, and stained with eosin and hematoxylin. Next, they were washed in tap water and allowed to dry^55^.

One hundred cells per slide were counted under an optical microscope with a 100x oil immersion objective, in order to differentiate cell types (macrophages, lymphocytes and neutrophils). The cell count in the joint lavage was obtained by calculating the percentage of each cell type (differential count) and number of leukocytes in the joint lavage fluid^55^. The results were expressed as number of cells × 10^3^/ml.

### Determination of cytokine levels

TNF-α, IL-1β, IL-6, IL-17 and IL-10 concentrations were determined using a commercially available enzyme-linked immunosorbent assay (ELISA), following the manufacturer’s instructions (Duo-Set kits; R&D Systems, Minneapolis, MN, USA). The optical density of the individual samples was measured at 450 nm using a spectrophotometer (Spectra Max-250, Molecular Devices, Sunnyvale, CA, USA). Results were expressed as the mean ± SD of cytokine levels in pg/mg of joint fluid^57,58^.

### Histopathological assessment of synovitis

Articular capsule samples were fixed in 4% (vol/vol) buffered formalin, dehydrated in ethanol, and embedded in paraffin for slide preparation. Tissue sections were stained with hematoxylin and eosin (H&E) to analyze synovitis (inflammatory cell influx and synovial hyperplasia). The severity of the synovial pathology (i.e., synovitis) was determined using a scoring system that measures the thickness of the synovial cell layer on a scale of 0–3 (0 = 1–2 cells, 1 = 2–4 cells, 2 = 4–9 cells, and 3 = 10 or more cells) and cell density in the synovial stroma on a scale of 0–3 (0 = normal cellularity, 1 = slightly increased cellularity, 2 = moderately increased cellularity, and 3 = greatly increased cellularity)^59^. Additional slides were stained using the Sirius Red staining protocol in order to evaluate articular fibrosis. Collagen deposition under the synovial membrane was measured as the Sirius Red-positive staining area in 15 random high power fields (400x magnification) using Image J software (Image J, 1.33 u, USA). The results are expressed as a percentage of collagen-positive area.

### Statistical analysis

The analyses were performed using *Statistical Package for the Social Sciences* software (SPSS 22.0 Inc, Chicago, IL). The homogeneity of variance and the normality distribution were checked using the Levene and the Shapiro-Wilk tests, respectively. Two-way ANOVA was performed for gait test, skin temperature, knee joint swelling and thermal response threshold, with group (Control, KOA, KOA + Cryotherapy and KOA + Placebo) and time (0, 60 and 66 days) interaction. One-way ANOVA was carried out to compare between group differential leukocyte count, cytokines levels and articular fibrosis. When necessary, Tukey’s HSD post-hoc test was performed [α = 5% and 95% confidence interval (CI)]. The synovitis score did not present a normal distribution, and they were analyzed using nonparametric tests. Kruskal-Wallis test was used to assess the synovitis between groups and Mann-Whitney test was used to identify the differences among groups if Kruskal-Wallis results indicated significant differences. For all nonparametric comparisons among groups using Mann-Whitney test, the alpha level was adjusted according to the number of comparisons (Control × KOA, Control × KOA + Cryotherapy, Control × KOA + Placebo, KOA × KOA + Cryotherapy, KOA × KOA + Placebo, KOA + Cryotherapy × KOA + Placebo) or α = 0.05/6 = 0.008. Thus, *P* value < 0.008 was regarded as statistically significant difference.

## Conclusion

Footprint patterns improved in rats with ACLT-induced KOA as a result of clinical-like cryotherapy, which also lowered the synovial fluid leukocyte count and inflammatory cytokine concentration in these rats. These findings demonstrate the benefits of cryotherapy, confirming its potential and as a non-pharmacological treatment for joint inflammation in the KOA.

## Supplementary information

Supplementary appendix I and II
