# Supplementary material for: Clinical-like cryotherapy improves footprint patterns and reduces synovial inflammation in a rat model of post-traumatic knee osteoarthritis
Source: Sci Rep. 2019 Oct 10;9:14518. doi: 10.1038/s41598-019-50958-8 (PMC6787208; doi:10.1038/s41598-019-50958-8)
Supplement: Supplementary file 1 — Supplementary appendix I and II [file 41598_2019_50958_MOESM1_ESM.docx]

**Clinical-like cryotherapy improves footprint patterns and reduces synovial inflammation in a rat model of post-traumatic knee osteoarthritis**

Germanna Medeiros Barbosa¹, PT, MSc; Jonathan Emanuel Cunha¹, PT, MSc; Thiago Mattar Cunha^2^, MSc, PhD; Lizandra Botaro Martinho^1^, PT; Paula Aiello Tomé de Souza Castro¹, MSc, PhD; Francisco Fábio de Oliveira^2^, MSc, PhD; Fernando Cunha^2^, MSc, PhD; Fernando Silva Ramalho^3^, MSc, PhD; Tânia Salvini¹, PT, MSc, PhD.

**Supplementary appendix I**

**
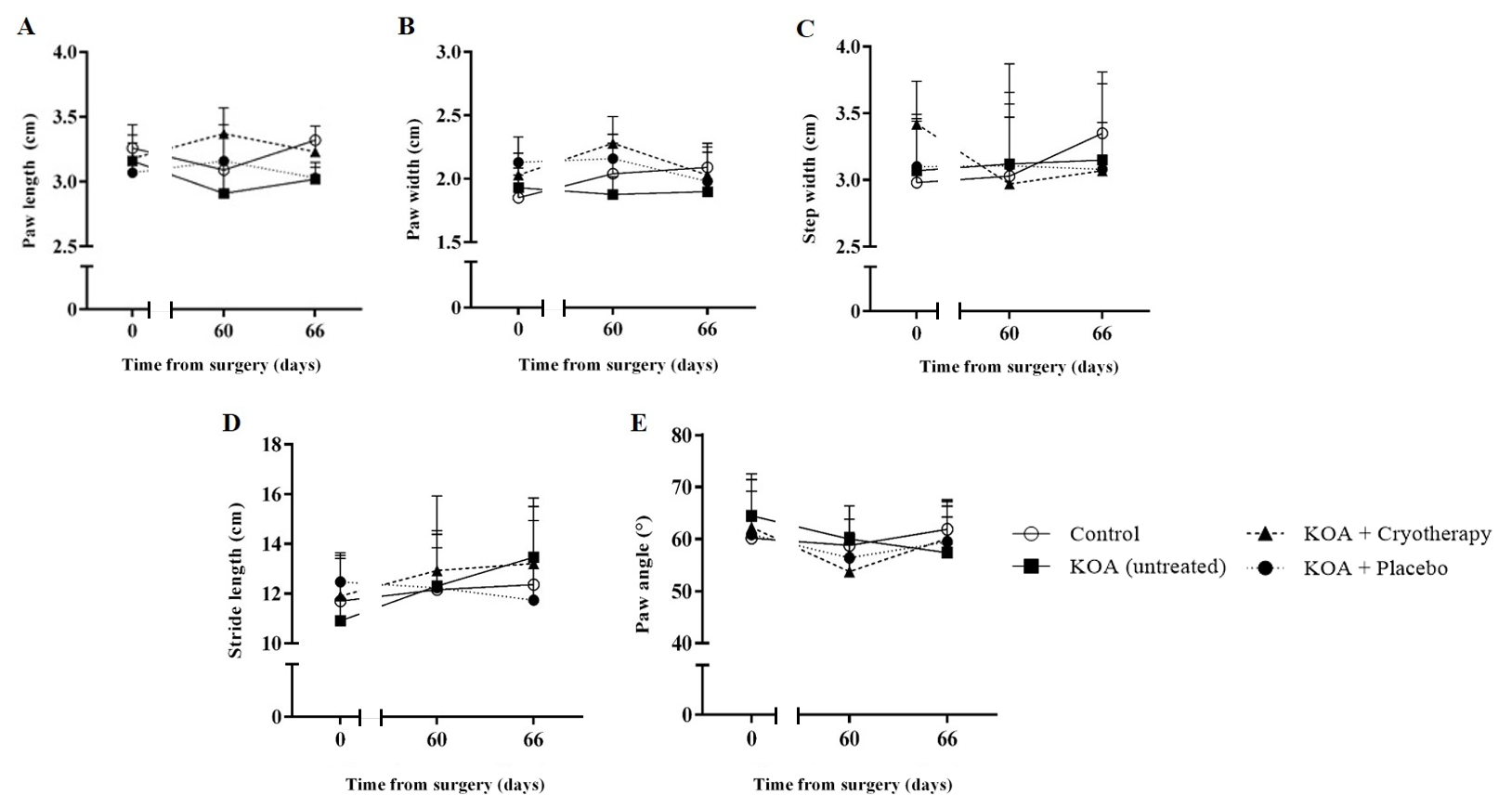
**

Gait pattern measured by the paw print test. A= paw length, B= paw width, C= step width, D= stride length, E= paw angle at (0 day), pre (60th day) and post (66th day) intervention protocol in all groups. KOA: knee surgery (anterior cruciate ligament transection). Data are expressed as mean ± SD (n= 8/group).

**Supplementary appendix II**

**
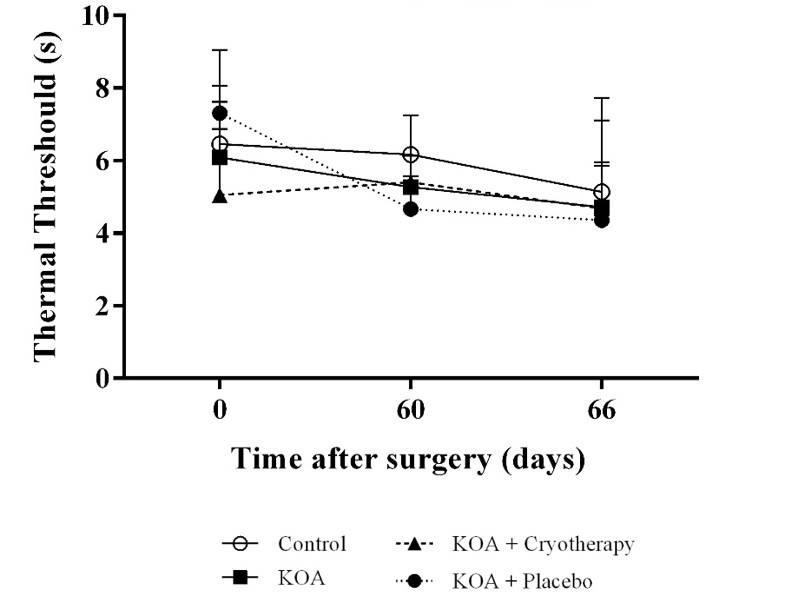
**

Thermal response threshold at baseline (0 day), pre (60^th^ day) and post (66^th^ day) intervention protocol in all groups. KOA: knee surgery (anterior cruciate ligament transection). Data are expressed as mean ± SD (n= 8/group).
